# Parental versus child reporting of fruit and vegetable consumption

**DOI:** 10.1186/1479-5868-4-33

**Published:** 2007-08-24

**Authors:** Evelien Reinaerts, Jascha de Nooijer, Nanne K de Vries

**Affiliations:** 1 Nutrition and Toxicology Research Institute Maastricht (NUTRIM), Department of Health Promotion and Health Education, Universiteit Maastricht, PO Box 616, 6200 MD, Maastricht, The Netherlands; 2 Care And Public Health Research Institute (CAPHRI), Nutrition and Toxicology Research Institute Maastricht (NUTRIM), Department of Health Promotion and Health Education, Universiteit Maastricht, PO Box 616, 6200 MD, Maastricht, The Netherlands

## Abstract

**Background:**

The purpose of this study was to (1) compare parental and child recording of children's fruit and vegetable (F&V) consumption, including family-related factors, and (2) investigate the potential differences in the relation of children's and parents' perceptions of family-related factors.

**Methods:**

Children were recruited from Dutch seventh and eighth grade classrooms. Each child and one of their parents completed parallel questionnaires. A total of 371 matched child-parent surveys were included in the analyses. To compare parental and child reports of consumption and family-related factors regarding F&V intake several techniques were used such as paired sample t-test, chi-square tests, Pearson's correlations and Cohens's kappa as measurement of agreement. To investigate potential differences between the parent's and children's perceptions of family-related factors, linear regression analyses were conducted.

**Results:**

The results indicated weak agreement for F&V consumption (Cohen's kappa coefficients of .31 and .20, respectively) but no differences in mean consumption of fruit at the group level. Regarding the family-environmental factors related to fruit consumption, significant differences were found between the perceptions of subjective norm, and the availability and accessibility of fruit. Perceptions of subjective norm, parental modelling and exposure regarding vegetable consumption were also viewed differently by the two groups. The family-environmental factors reported by the children were similarly associated with F&V consumption compared to those reported by their respective parents. However, parents rated these factors more favourably than their children did.

**Conclusion:**

The results indicated a low level of agreement between parental and child reporting of F&V intake and their assessment of family-environmental factors on individual level. This has important implications for the development and evaluation of interventions and we recommend that researchers clearly indicate which source of information they use in their studies as it remains unclear which source is more valid.

However, when the effects of interventions are studied at the group level, our results suggest that it makes no difference whether children or parents report the child's fruit consumption. The same holds for determinant studies; both parental and child reports can be used. However, perceptions of these factors differ significantly.

## Background

Since the World Health Organization adopted a worldwide strategy making the promotion of a healthy diet a priority in public health policy [1], there has been an increase in the number of initiatives undertaken to promote healthy eating habits such as the daily consumption of sufficient amounts of fruit and vegetables (F&V). Many of these initiatives focus on children [2] because adult eating habits are acquired during childhood [3,4] and children are more apt to change their dietary patterns [5].

Recently, several primary school programmes have been developed to increase the poor consumption of F&V [6] among Dutch children aged 4 to12 [7,8]. The social ecological perspective [9] assumes that the effectiveness of interventions can be enhanced significantly through the coordination of individuals acting at different levels [10], such as parents providing their children with an environment, that supports F&V consumption. Parents can increase the availability and accessibility of F&V at home, and thereby reinforce and model F&V intake (see Nicklas et al., 2001 [11] for a review). They can even shape their children's taste preferences by repeatedly exposing their children to F&V [12,13].

Two recent reviews demonstrated the importance of these family-related factors by concluding that taste preferences and the availability and accessibility of F&V were the most influential determinants of primary school children's F&V intake [14,15]. Parental behaviour, modelling and feeding practices were also identified as relevant determinants of F&V consumption in children [14].

Research has shown that children often perceive family-related factors differently than their parents [16-18]. Insight with respect to these differences is essential given their implications for intervention development and evaluation. Problems often occur when perceptual differences lead to a lack of parental motivation to create an environment that supports sufficient F&V consumption by children. For example, a parent may report being supportive by making sufficient F&V available in the home, while their children report a lack of readily available fruit at home. To ensure programme effectiveness, health promotion planners may have to include strategies in their interventions to target these perceptual differences.

A second implication relates to the interpretation of effect studies. Interventions directed at primary school children are often evaluated using parental reporting, because it is generally thought that younger children are limited in their ability to self-report their food intake [19]. However, studies have shown that neither children [19-21] nor parents [22] are always reliable reporters of a child's food intake. Since no objective measure of F&V consumption is available, investigating the differences in perception of intake between these two groups is important.

Our literature review found only two studies that addressed the lack of consensus between child and parental reporting for primary school children. A Dutch study showed low levels of agreement regarding F&V intake and a limited set of environmental factors including perceived variety of consumption, availability of F&V and several food rules at home [17]. In this study, the fourth grade children self-reported a higher F&V intake compared to their parent's assessment. The level of agreement between perceptions of environmental factors was higher than the level of agreement for consumption measures. However, both were still relatively low. Despite these low levels of agreement, it was concluded that, in the absence of an objective measure of F&V consumption, parental reporting could be a valid method for measuring children's F&V intake since the results were supported by previously reported intake levels in the Netherlands.

The second study was aimed primarily at identifying correlates of children's F&V intake. However, it also compared parents' and children's reporting of children's F&V accessibility, skills and preferences [16]. The results demonstrated that parent-child correlations of these constructs were moderate (.30< r <.35). They also indicated that parents perceived higher levels of F&V accessibility at home than their children did. Additionally, parents thought their children had less behavioural skills than the children reported having [16]. This study concluded that, since parents control the home food environment and since differences in perceptions occur, interventions are needed that are directed at the parents.

In the absence of a gold standard or an objective measure of F&V consumption, no conclusions can be drawn as to whether children's or parents' reporting is more valid. Therefore, to properly interpret the effectiveness of interventions, insight on the lack of agreement between child and parental reporting remains essential. Furthermore, additional insight on differences relating to family-environmental constructs and F&V-consumption between parents and children is imperative.

Supplementary to the studies of Tak (2006) and Bere (2004), the current study also looked into the relation of children's and parents' perceptions of family-related factors. Although the relationship between adolescents' perceptions of family food rules and availability [18] has recently been studied, this relation in younger children has not. The perceptions of younger children may be even more significant than the perceptions of older children, given that, for younger children, the family has a greater influence on food access and intake [23]. Therefore, the purpose of study reported here was to (1) compare parental and child recording of children's fruit and vegetable (F&V) consumption, including family-related factors, and (2) investigate the potential differences in the relation of children's and parents' perceptions of family-related factors. The possible implications of these results for intervention development and the interpretation of programme effectiveness are also discussed.

## Methods

### Respondents and procedures

We used data from the baseline measurement of an intervention study [8]. The Regional Health Service invited every school in the middle and north region of the province of Limburg, which had at least 200 students (n = 49) to participate in the current study. Of these 49 schools, 12 (25%) agreed to participate. Although non-participation was not studied extensively, lack of time was the most mentioned reason not to participate. These reasons were demonstrated previously in both national and international literature [24-26]. Parents were recruited through an information sheet the children took home and informed consent was acquired.

The study presented here focused only on children in the seventh and eighth grade and their parents, since children of this age were considered capable of accurately filling out questionnaires [19]. The parental questionnaires were given to the children to take home and, once completed, were collected by the teachers. The teachers were also responsible for administering the questionnaires to the children in the classrooms. Of the 486 children for which informed consent was obtained, 423 children (87%) filled out a questionnaire in the classroom and 371 of the parents returned their parental questionnaire. A total of 371 matched child-parent pairs were thus assembled for analyses. The 13% non-response rate among the children was due to their absence at the time the questionnaire was administered.

### Questionnaires

Two similar questionnaires that included parallel indicators of children's F&V consumption and potential correlates of intake were used. The parental questionnaire had been used in a previous study and is described more extensively elsewhere [27]. Parents were instructed that the questionnaire had to be completed by the parent that usually takes care of what the child eats. Parallel *demographic indicators *included questions regarding the child's weight, height, age, gender and ethnicity. Using the Statistics Netherlands definitions of native and non-native residents, the children were classified as being 'native' when both parents were born in the Netherlands and as 'non-native' when one parent had been born outside of the Netherlands [28]. The parental questionnaire included additional questions regarding the parent's gender, education level and their F&V consumption, using a validated 10-item questionnaire [29].

The *family- environmental factors *measured included modelling, subjective norm, exposure, accessibility and availability. These were assessed separately for F&V consumption using the same format for both F&V. Table 1 presents the number of items, range, Cronbach's α, and examples of items.

**Table 1:** Description of family- environmental factors, numbers of items, mean (SD), internal consistency, sample items and range

	No. of items	Internal consistency (α)		Examples of questions, response options and ranges	
		Child	Parent		

*Fruit consumption*					
Modelling by mother	1	-	-	My child's mother eats fruit everyday; totally disagree (-2) to totally agree (+2)	
Modelling by father	1			My child's father eats fruit everyday; totally disagree (-2) to totally agree (+2)	
Subjective norm (nb * mc)	2	-	-	*Motivation to comply *(mc): My child does what we tell him/her to do; totally disagree (1) to totally agree (5) *Normative belief *(nb): My child thinks we want him/her to eat more fruit; totally disagree (-2) to totally agree (+2)	
Exposure	14	.72	.71	Has your child ever tasted tangerines, banana, kiwi fruit, etc? (0–14)	
Accessibility	1	-	-	Do you or your partner (sometimes) prepare fruit for your child? Yes (1) or no (0)	
Availability	2	.51	.73	[1] Do you always have fruit [2] that your child likes available at home? Yes, always (+2) to No, never (-2)	

*Vegetable consumption*					
Modelling by mother	1	-	-	My child's mother eats vegetables everyday; totally disagree (-2) to totally agree (+2)	
Modelling by father	1	-	-	My child's father eats vegetables everyday; totally disagree (-2) to totally agree (+2)	
Subjective norm (nb * mc)	2	-	-	-	
Exposure	15	.67	.78	Has your child ever tasted cauliflower, broccoli, carrots, etc? (0–15)	
Accessibility	1	-	-	Do you or your partner (sometimes) prepare vegetables as a snack for your child? Yes (1) or no (0)	
Availability	2	.49	.57	[1] Do you always have vegetables, [2] that your child likes available at home? Yes, always (+2) to No, never (-2)	

Children's *fruit consumption *was assessed using two questions: (1) 'How many days per week does your child eat fruit/do you eat fruit ?' (Answers ranged from one to seven days); and (2) 'How many portions of fruit does your child eat/do you eat on a day that he or she/you consume(s) fruit?' (Answers ranged from '1/2 portion a day' to '3 portions a day or more' on a six-point scale). The average consumption of whole fruit (in portions per day) was calculated by multiplying both questions and dividing the result by seven.

Children's *frequency of vegetable intake *was measured using three questions:(1) 'How many times per week does your child eat cooked or baked vegetables for dinner (including mixed dishes)?'; (2) 'How many times per week does your child eat mixed dishes like macaroni?'; and (3) 'How many times per week does your child eat extra salad items, like lettuce, tomato, or other raw vegetables?' The number of days that the children consumed cooked vegetables was calculated by subtracting mixed dishes from cooked or baked vegetables, including mixed dishes.* Portion size *was assessed using photographs of plates filled with different amounts of cooked vegetables (25-50-100-150 grams) or mixed dishes (75-150-300-450 grams). Respondents were asked to select the photograph that best represents the amount of food that the child usually consumes. According to the Netherlands Nutrition Centre, on average, 33% of a mixed dish consists of vegetables [30]. The amount of extra salad or raw vegetables was calculated by multiplying frequency per week by 35 grams (the weight of a small bowl of salad). Lastly, *the average consumption of vegetables in grams per day *was computed as follows: ((the number of days that the children consumed cooked vegetables * portion size) plus (the number of days children ate mixed dishes * (.33 * portion size) plus (the number of days children ate extra salad or raw vegetables * 35 gram))/seven days.

The FFQ method was used in a similar Dutch project [7] and was based on the Pro-children questionnaire that was validated by Haraldsdóttir and colleagues [31].

### Data analysis

Means, standard deviations and percentages were used to describe consumption and family-related factors. The consumption measures and continuous family-related variables were checked for normality. The F&V consumption measures showed positively skewed distributions (Z_skewness _> 2), and all of the family-related factors showed negatively skewed distributions (Z_skewness _< -2). Therefore, results from both non-parametric tests are reported for both variables. To compare parental and child recording of the child's F&V consumption and family-related factors regarding F&V intake (research question 1), several techniques were used. Wilcoxon signed-rank test and chi-square tests were used to assess differences in means for the intake measures and family-environmental factors. Spearman's correlation coefficients were used to assess associations between parental and child recording. Furthermore, we assessed how many parents reported higher, equal, or lower consumption compared to their children's reports by dividing the consumption levels reported by both child and parent into four equal groups (see Table 2 for a description). We also calculated Cohen's kappa coefficients as a measure of agreement between parental and child reporting. Separate logistic regression analyses were used to study associations between agreement and parent or child characteristics. We used underestimation (1) versus equal estimation (0) of consumption as the dependent variable and the child's gender, ethnicity, age and BMI, and parent's gender, education, F&V consumption and the child's level of F&V consumption (below median of averaged parent and child report versus above median of averaged consumption) as independent variables. The same analyses were conducted for overestimation (1) versus equal estimation (0). The analyses were carried out separately for fruit and vegetable consumption. All analyses were performed using SPSS 13.0.

**Table 2:** Number of respondents, means (SD) and range within each quartile, separately for fruit and vegetable consumption

	Quartile	n	Mean (SD)		Range	
		child/parent	Child	Parent	Child	Parent
*Fruit consumption*	1	84/88	.25(.17)	.27(.16)	.00–.50	.00–.43
*(portions per day)*	2	103/121	.71(.11)	.69(.11)	.57–.86	.57–.86
	3	95/72	1.13(.16)	1.14(.16)	1.00–1.44	.94–1.43
	4	89/90	2.05(.48)	1.98(.42)	1.50–3.00	1.50–3.00
*Vegetable consumption*	1	102/98	32.53(13.27)	35.55(12.20)	.00–50.00	.00–50.00
*(grams per day)*	2	84/91	60.70(5.22)	58.61(4.02)	50.71–70.00	50.71–65.00
	3	94/90	82.66(7.76)	73.15(4.84)	71.16–97.86	65.45–83.57
	4	91/92	121.49(19.53)	107.74(18.58)	98.28–185.00	84.29–175.00

To investigate potential differences between the parent's and children's perceptions of family-related factors (research question 2), multi-level regression analyses [32] were conducted, extending the fixed regression model with a random school effect. For these analyses the F&V consumption reported by the child was used as the dependent variable and these were adjusted for positive skewness using square root transformations (SQRT(X)). First, the associations of family-related factors with child-reported F&V intake were studied in separate analyses using child versus parental reporting of the family related factors (modelling, subjective norm, exposure, accessibility, and availability). The indicator for *accessibility *of vegetables that focused on vegetable snack consumption (e.g. cucumber, carrot sticks, etc) was excluded from the analyses because too many respondents indicated that their child never consumed vegetables as a snack. Secondly, we tested whether the regression coefficients of parental and child reported family-related factors differed significantly. To do this, multilevel regression analyses with F&V consumption reported by the child as the dependent variable were conducted. Predictors in these analyses were the family-environmental variables, a dummy variable that was coded 1 for child reporting and 0 for parental reporting, and a variable that was the product of these two variables.

## Results

Of the total sample of children, 47% was male, 53% was female and most were of Dutch origin (72%). On average, the children were 11.0 (Standard Deviation 0.8) years old, ranging from 9 to 13, and the mean BMI was 17.5 (SD 2.9), indicating a normal weight according to international standards for children [33]. The parental questionnaires were completed most often by the mother (83%) and the parents' mean age was 41.3 (SD 5.0). Of the parents, 33% had a low level of education, 52% a medium level of education and 15% had a high level of education. The parents consumed about 2.5 (SD 2.5) portions of fruit, including fruit juice, and about 3.3 (SD 1.6) tablespoons of vegetables per day (the equivalent of approximately 165 grams per day).

### Comparison between parental and child recording of children's F&V consumption

Almost half of the child-parent pairs were in the same quartile for fruit consumption (Table 3), 37% were in an adjacent quartile and 15% in a different quartile (see appendix 1). About 40% of the pairs were in the same quartile for vegetable consumption, 36% were in an adjacent quartile and 24% were in a different quartile (see appendix 1). Cohen's kappa's for F&V consumption were significant but low, with .31 and .20, respectively. As shown in Table 4, parents' and children's reported intake of the fruit consumption of the child did not differ. However, children did report their own vegetable consumption significantly higher than parent's reported (p < .01). Correlations between parent and child reporting of the child's consumption were .55 (fruit consumption) and .39 (vegetable consumption), indicating a large and moderate correlation [34].

**Table 3:** Estimation of F&V consumption by parents using child as reference and quartiles

	Lower quartile compared to child n (%)	Same quartile as child n (%)	Higher quartile compared to child n (%)	Cohen's kappa
*Fruit consumption*	105(28.3)	179(48.2)	87(23.5)	.31***
*Vegetable consumption*	109(29.4)	148(39.9)	114(30.7)	.20***

* p < .05; **p < .01; ***p < .001

**Table 4:** Parent-child comparisons and standardized regression coefficients between children's F&V intake, and family-environmental constructs

	N (pairs)	Mean (SD)		p-value for difference in group frequency or means^a^	Spearman's correlation	β consumption	
		Child	Parent			Child	Parent

*Fruit consumption (portions per day)*	371	1.03(.70)	.99(.67)	.31	.55***		
Modelling by mother	319	.97(1.04)	.93(1.15)	.89	.39***	.04*	.02
Modelling by father	301	.84(1.23)	.81(1.30)	.48	.48***	.02	.03
Subjective norm (nb * mc)	371	-.29(5.67)	2.59(4.95)	.00	.28***	-.01***	-.02**
Exposure	371	11.98(1.74)	11.85(1.75)	.13	.52***	.04***	.03**
Accessibility^b^	337	51%	69%	.00	.47***	-.03	-.01
Availability	371	1.46(.57)	1.56(.56)	.00	.40***	.13***	.09*
*Vegetable consumption (grams per day)*	371	73.4(35.4)	68.2(28.9)	.01	.39***		
Modelling by mother	350	1.60(66)	1.74(.57)	.00	.27***	.38	.19
Modelling by father	336	1.54(.75)	1.63(.73)	.01	.28***	-.15	-.17
Subjective norm (nb * mc)	364	-.55(5.68)	2.19(5.27)	.00	.28***	-.07***	-.07**
Exposure	371	13.07(1.74)	13.19(2.10)	.00	.50***	.16*	.05
Accessibility^b^	140	61%	70%	.30	*.08*	-	-
Availability	371	1.36(.60)	1.41(.54)	.16	.27***	.61**	.40

^a^Wilcoxon signed rank tests were used to compare average consumption of parent and child; ^b^Due to dichotomized character of variable relative percentages of accessibility (% yes) are presented together with chi-square test and kappa's measure of agreement; * p < .05; **p < .01; ***p < .001.

Logistic analyses showed a significant association between underestimation of fruit consumption and ethnicity. In comparison to parents of native children, parents of non-native children more often reported lower fruit consumption than their children (OR 2.19, 95%CI: 1.19–4.11). Results also showed a significant association between overestimation of fruit consumption and child's BMI and fruit consumption level. Parents of children with a high BMI (OR 1.12; 95%CI: 1.01–1.23) and parents of children that consume higher amounts of fruit (OR 2.69; 95%CI: 1.49–4.95) reported more often higher fruit consumption than their children. Furthermore, parents of children that consumed higher amounts of vegetables reported a lower vegetable intake for their child (OR 1.98; 95%CI: 1.14–3.44), compared to the child report.

### Family-environmental factors and associations with F&V consumption

Table 4 displays the differences between the parental and child reporting of subjective norm, accessibility, and availability of fruit, as well as modelling by mother and father, subjective norm and exposure regarding vegetable consumption. Parents reported more positively on all these constructs than their children. Correlations between parent and child were significant but mostly low to moderate, ranging from .27 to .52 (p < .001).

Subjective norm, exposure, and availability were significantly associated with fruit consumption with both parents and children. Subjective norm and availability were significantly correlated for vegetable consumption in parental and child measures. Only in the children's reports was exposure also correlated with vegetable consumption. No statistical differences between regression coefficients of the parents and the children were found, indicating similar associations.

## Discussion

This study compared parental and child reporting of F&V consumption and family-related factors. Our results demonstrated a low level of agreement between child and parental reporting for both F&V intake. These results are comparable to the results of a similar study [17]. However, we must conclude that, contrary to previous findings, both agreement and correlation between child and parental reporting are better for fruit consumption than for vegetable consumption. This could be due to the sensitivity of the measurements. To evaluate fruit consumption, we asked to report the number of servings per day. To evaluate vegetable consumption, we asked the respondents to report portion sizes (in grams). A study by Frobisher [35]demonstrated that parent and child estimations of portion size, even when using photographs, are often inaccurate. This could account for the lower level of agreement for vegetable consumption. Furthermore, we found similar numbers of parents reporting higher estimates of their child's consumption compared to their child's reports, as the number of parents that estimated lower levels of consumption when compared to their respective children.

However, our results showed that parents of high consumers more often perceived their child's fruit intake higher, and their child's vegetable intake lower, compared to the child report. In line with our results, Tak et al. [17] showed that high consumers of both fruit and vegetables had poorer levels of agreement than low consumers. This is perhaps due to a higher range in intake levels among the high consumers and may thus indicate a floor effect among the low consumers. Our results showed that parents of non-native children perceived their child's fruit intake lower than their child did. This is surprising, considering that the non-native children consumed more fruit compared to native children (p < .05) (see appendix 2). An earlier study among children in the Netherlands also showed that non-native children are among the high consumers [36] and based on this one would expect that parents of non-native children therefore should 'overestimate' their child's intake. Tak et al. [17] also found mixed results regarding level of agreement and ethnicity, so further study into this relation is needed. The finding that parents of children with a higher BMI perceived their child's fruit intake to be higher might be caused by the fact that parents could feel responsible for their child's weight status and therefore report in a more social desirable way.

Regarding family-environmental factors, our results indicated that child and parental reporting of fruit consumption differed significantly for subjective norm, accessibility and availability. Moreover, with respect to vegetable consumption, differences were found for subjective norm, exposure and modelling of vegetable consumption by parents. Combined with the low to moderate correlation between the reporting of both groups, we can conclude that perceptions of important family-environmental factors differ between children and their parents. These results are in accordance with previous studies [16,18] and are thus cause for concern. Since parents perceived these environmental factors more positively than their children did, they may believe that they are creating a supportive environment and therefore see no need to change the home environment in a way that can facilitate their children's consumption of F&V. Their children, conversely, perceive the environment to be less supportive. This is especially important for program development, because the majority of interventions targeting children include a parental component [2,37,38]. Unfortunately, the situation described above could hinder the implementation of strategies aimed at parents and thereby limit the effectiveness of interventions.

The second objective of the study was to examine the relationship between family-related factors and F&V intake. We found that subjective norm, exposure and availability were important correlates of fruit consumption in both child and parental measures. Subjective norm and availability were significantly correlated with vegetable consumption for both parallel scales but exposure was only identified as a correlate when the child's reports were used. Interestingly, no differences in the association of family-related factors with F&V consumption between child and parent reports were detected. To date, only one similar study was found that focused on adolescents and their parents [18]. As in our study, the researchers did not find differences in the relationship between accessibility or availability and fruit consumption.

The most important limitation of studies that compare intake based on reporting by children and their parents is that determining whether the child's or parent's report is the more valid measure is impossible. There is no objective measure with which these reports can be compared. It is also important to note that this study focused on a limited number of family-environmental factors. More and more research indicates the importance of parenting practices, like food rules, and parenting styles that foster a healthy lifestyle [18,39,40]. Consequently, we contend that additional research on how the role of parenting can generate healthy nutrition of children is necessary. Finally, we used one or two items to measure most family-environmental factors. However, these measures can be considered if the item reflects a homogeneous construct [41-43]. Although these items are common in comparable studies (e.g. [44]), single-item measures usually have a low reliability. Therefore, multiple-item measures are still more desirable, but the time required to fill out the questionnaire limited the use of these measures.

In sum, we found a low level of agreement between parental and child reporting of both fruit and vegetable intake at the individual level. For empirical purposes, we recommend that researchers clearly indicate which source of information they use in their studies as it remains unclear which source is more valid. A combination of both kinds of reporting in effect studies is preferred. However, when the effects of interventions are studied at the group level, the lack of differences in mean fruit consumption and the large correlation between parental and child reporting suggest that it makes no difference whether children themselves, or parents as a proxy, report the child's fruit consumption.

The same holds for determinant studies. When looking at important correlates of F&V consumption, one can conclude that the same family-environmental factors appear to be important independent sources of reporting. Thus, when conducting determinant studies, both parental and child reports can be used. However, perceptions of these factors differ significantly. This has important practical implications for intervention development. Those who develop interventions must take these differences in perception into account and also endeavour to inform parents about these discrepancies.

## Conclusion

We conclude that parental and child reporting of both fruit and vegetable intake show low levels of agreement on the individual level, but are acceptable for fruit consumption studied at the group level.

Furthermore, independent of source of reporting, the same family-environmental factors are important regarding F&V intake. However, perceptions of these factors differ significantly between parent and children.

## Competing interests

The author(s) declare that they have no competing interests.

## Authors' contributions

ER collected and analysed the data and drafted the manuscript. JdN and NdV participated in the study design and provided critical revision of the manuscript. All three authors have read and approved the final manuscript.

## Appendix

^1 ^This data is not included in the table but can be obtained from the author upon request.

^2 ^Data not published.
